# ‘Some Days Are Not a Good Day to Be a Mum’: Exploring Lived Experiences of Guilt and Shame in the Early Postpartum Period

**DOI:** 10.3390/ejihpe14120198

**Published:** 2024-12-02

**Authors:** Leanne Jackson, Emily O’Donoghue, Jasmin Helm, Rita Gentilcore, Anisha Hussain

**Affiliations:** Department of Psychology, Institute of Population Health, University of Liverpool, Liverpool L69 7ZA, UK

**Keywords:** guilt, shame, morality, postpartum, parenting, body image, embarrassment, stigma, mental health, intensive mothering theory

## Abstract

The first 16 weeks postpartum are particularly challenging for a new mother and are associated with an elevated risk of experiencing psychological distress. Guilt and shame have been identified as significant predictors of other forms of psychological distress, such as anxiety and depression. However, guilt and shame are poorly distinguished in pre-existing literature. The current study used inductive thematic analysis to explore lived experiences of guilt and shame in the early postpartum period. Semi-structured interviews were conducted with 20 women who had given birth in the past 16 weeks and who were residing in the UK at the time of the investigation. All those interviewed had internalised unrealistic mothering ideals. Physical constraints on one’s parenting abilities, due to birth recovery, exacerbated postpartum guilt and shame. Other sources of guilt and shame included body dissatisfaction and making comparisons against other mothers and media depictions of motherhood. Participants were hesitant to confide in others about parenting challenges due to fears of judgement, which perpetuated the shame-concealment cycle. Future research should prioritise the development of interventions designed to target a harsh parenting inner critic, and the re-framing of cognitive biases, to nurture more realistic and self-compassionate beliefs about motherhood. For practice, current findings mirror previous calls for intimate partners to be actively included in routine appointments, to provide healthcare practitioners with specialist training in postpartum mental health, and to educate mothers on responsible social media use.

## 1. Introduction

The first 16 weeks postpartum are particularly challenging for a new mother, representing a steep learning curve for acquiring parenting skills and managing newfound responsibilities [1]. Coupled with hormonal changes in the early postpartum [2], the risk of experiencing emotional distress is particularly elevated in the first 16 weeks after giving birth [3]. Despite idealised portrayals on social media [4], new motherhood also encompasses feelings of overwhelm, irritability, and parental burnout [5]. Prevalence of postpartum anxiety and depressive disorders in the early postpartum stand at 11.1% and 6.1%, respectively [6,7]. Postpartum psychological distress negatively impacts mother–infant bonding and child development outcomes [8] and is associated with poorer maternal quality of life [9]. Deteriorated quality of life also has adverse economic consequences in terms of NHS treatment costs and indirect workplace costs due to periods of absence [10]. It is, therefore, of utmost importance to identify and address factors that lead to the onset and maintenance of psychological distress during this critical period.

Recent literature evidence guilt and shame as significant predictors of postpartum depression and anxiety symptoms [11,12,13]. Guilt is defined as feelings of remorse concerning a moral transgression, while shame involves the internalisation of said guilt to the self as a global entity [14]. Unlike guilt, which is behaviourally tied, shame is a self-directed moral emotion [15]. In a parenting context, guilt stems from feeling defensive or selfish about one’s perceived shortcomings, while shame manifests when perceived parental failings are judged harshly by others [16]. This results in self-blame, self-abandonment, aggression, insecurity, and help-seeking avoidance [17,18].

New mothers are subject to the mothering myth: a cultural tool that perpetuates unrealistic expectations to be a constant nurturing presence for one’s infant [19,20]. This contributes to feelings of guilt and shame when new mothers inevitably fall short of such unattainable standards [21]. Common sources of parenting guilt and shame include spending time away from one’s child(ren) [22], infant feeding challenges [23], concern for infant welfare [24], declined relationship satisfaction [25], concerns about body image [26], and physical complaints, e.g., fatigue [27]. Sleep quality is poorest in the first three months postpartum, which can further exacerbate emotional and psychological issues [28]. Poor sleep quality can also adversely affect mother–infant bonding [29]. Certainly, the early postpartum appears to be a critical period for psychological adjustment to new motherhood and for the onset of psychological distress.

Despite their omnipotence, however, guilt and shame are often conflated in parenting research [23], which renders conceptual differentiation difficult [30,31]. This is problematic because guilt and shame are characterised by distinct antecedents, attributes, and consequences [16]. For example, shame indirectly (and more strongly) predicts depression and anxiety symptoms through feelings of dejection from one’s social and healthcare professional network, while guilt indirectly predicts depression and anxiety through the infant feeding method [13]. Guilt and shame are considered as transdiagnostic phenomena, both contributing to the onset and maintenance of various forms of psychological distress [11]. Gaining a deeper understanding of these emotions is therefore important for adopting a preventative approach to maternal mental health care. By better understanding lived experiences of sub-clinical emotions such as guilt and shame, it may be possible to enhance postpartum psychological wellbeing, overall quality of life, and prevent the onset of life-disruptive forms of distress, such as postpartum anxiety and depression. To the principal investigator’s knowledge, no previous research has explicitly focused on exploring the lived experiences of parenting-specific guilt and shame in the first 16 weeks postpartum. The current study aimed to address this research gap using semi-structured interviewing, analysed using inductive thematic analysis.

## 2. Materials and Methods

The current study is reported in line with Consolidated Criteria for Reporting Qualitative Research (COREQ) guidelines [32] and Standards for Reporting Qualitative Research (SRQR) guidelines [33].

### 2.1. Ethics Statement 

Ethical approval was granted on 11 October 2022 by the University of Liverpool Research Ethics Committee (Project ID: IPHS/11347).

### 2.2. Reflexivity Statement 

LJ had 10 years of experience in conducting sensitive qualitative research with perinatal populations at the time of the investigation, which fed into the training provided to student investigators ahead of commencing recruitment. LJ’s experience also aided with bracketing pre-conceived expectations, which enabled data analysis to be systematic and data-driven. At the time of investigation, LJ had the following credentials: BSc (Hons) Psychology, PhD, and Fellow of the Higher Education Academy. All members of the research team were cis female. Also, at the time of investigation, EO, JH, and RG were MSc Psychology students, while AH was a second-year BSc Psychology student. EO and JH both completed their BSc (Hons) Psychology degree, and RG completed an undergraduate degree in Counselling. All student investigators were full-time students at the University of Liverpool at the time of the investigation.

For all student investigators, the current study was their first experience with interviewing perinatal populations. EO and RG’s backgrounds in Cognitive Behavioural Therapy mentoring and in counselling, respectively, facilitated rapport-building, empathic active listening, protection of participant confidentiality, and the upholding of professional boundaries. The position of JH as a childless researcher was viewed as a particular advantage in maintaining a stance of objectivity. AH found that the close relationships held with new mothers in her personal life allowed for a deeper understanding of motherhood to be achieved. The potential for bias was lessened as assumptions were bracketed through memo writing and extensive reflective notes during analysis. Additionally, the interview schedule was constructed with careful consideration to ensure that questions were open and non-leading.

### 2.3. Design and Procedure

A cross-sectional qualitative design was used, and individual semi-structured interviews were conducted via telephone or video conference calling software, e.g., Zoom. Semi-structured interviews allowed researchers to address the pre-defined research aim, while offering flexibility for participants to share unanticipated, salient experiences. An interview schedule was developed, which was divided into three distinct sections: demographic information (specifically, maternal age, infant age (in weeks), highest level of education, occupational status, marital status, and ethnicity); defining postnatal guilt and shame for their stage of new motherhood; and discussing personal experiences of guilt and shame at their stage of new motherhood (see Appendix A for interview schedule). The interview schedule was not piloted prior to commencing study interviews. However, the interview schedule did not require revision during the process of interviewing as both questions and prompts were found to appropriately facilitate conversations with participants. Indeed, all interview schedule questions and prompts had a logical chronology, and comprehensively explored experiential guilt and shame in the early postpartum period.

### 2.4. Data Collection and Analysis 

Eligible participants were 18 years of age or older, English-fluent, UK residents, with a healthy infant (defined as >34 weeks’ gestation, weight >2500 g at birth, no clinical abnormalities, which would otherwise contradict breastfeeding ability, e.g., Human Immunodeficiency Virus [HIV] positive status), aged 4–8 or 12–16 weeks, and no current or historic diagnosis of a mental health condition. These criteria were excluded to avoid potential confounders, e.g., [34]. All participants gave informed electronic and audio-recorded consent and were verbally and electronically debriefed within 24 h of the interview taking place. Interview duration ranged from 25 to 55 min (MTime = 36 min) [EO, JH, RG, and AH].

### 2.5. Participant Characteristics

Purposive sampling was employed [35] to recruit 20 women who were 4–8 weeks or 12–16 weeks postpartum. This methodological decision replicated recent postpartum literature [36] and was deemed appropriate for achieving data saturation [37]. Recruitment was targeted to improve sample heterogeneity. Specifically, due to a large proportion of breastfeeding women recruited in early interviews, formula and combination feeding parenting groups on social media platforms were approached later in recruitment. Study advertisements were placed on social media platforms, e.g., Instagram, X, Facebook, and by word-of-mouth. Recruitment spanned November 2022 to March 2024. Eligible women self-selected to participate by clicking on an active survey link contained within study advertisements. The active survey link re-directed the mother to a Qualtrics survey, where contact details were invited. After 48 h of expressing interest, a student investigator made initial contact to confirm eligibility and arranged a time and date for interviewing [EO, JH, RG, and AH]. As such, there was no known relationship between the research participant and the research team prior to volunteering to take part in the current study.

After consenting to participate and agreeing on a date for interviewing, the participant’s email address was confirmed on the initial contact call, and an electronic information sheet and consent form were sent for the mother to read and sign prior to the interview date. All front-facing participant documentation clearly stated the study purpose, aims, and data storage, management, and usage policies. Participant front-facing documentation can be made available on reasonable request to the principal investigator [LJ]. Details regarding the student investigator’s characteristics were not disclosed to the participant during the current study in recognition of the participant as the knowledgeable expert and focus of the investigation. Participants were verbally reminded of the study’s aims and purpose prior to the interview commencing. All participants gave audio-recorded consent at the time of the interview, as recognition of consent as a continuous process.

### 2.6. Data Analysis

To address the study aims, the analysis focused on exploring the lived experiences of parenting-specific guilt and shame in the first 16 weeks postpartum. Analysis of participant’s self-reported definitions of guilt and shame was beyond the scope of the study’s research aim. Interviews were conducted by student investigators at one timepoint [EO, JH, RG, and AH]. Each interview was only attended by a research participant and a delivering student investigator to provide a private and relaxed environment for the participant to share their lived experiences. Interviews were audio-recorded using a Dictaphone. During interviews, field notes were taken so that the student investigators could bracket any emerging pre-conceptions about potentially salient interview points and could ask follow-up questions about the participant’s lived experiences. After all interview schedule questions had been asked, participants were given the opportunity to talk about anything else that was important to them and their lived experience of postpartum guilt and shame. Two weeks after the interview concluded (this period was determined to allow the participant the sufficient opportunity to withdraw from the current study post-interview, if they desired), audio recordings were transcribed (verbatim) using NVivo 12 and Microsoft Word [EO, JH, RG, and AH]. Interview transcripts were not returned to participants for comment and/or correction. 

The current study adopted a social constructivist epistemology [38]. Social constructivism believes that psychological phenomena, one’s identity, and meaning-making are inherently tied to one’s sociocultural context [38]. This epistemological approach well aligned with the study’s research aim, which sought to better understand the lived experiences of guilt and shame in the first 16 weeks postpartum, for a UK sample of women. This approach recognises the importance of context in conceptualising, defining, and discussing complex emotions such as guilt and shame [39].

An idealist ontology was also an adopted lens for the current study [40]. Idealism recognises that the mind plays a central role in the construction of reality and that psychological phenomena constitute reality in and of itself [40]. Adopting an idealist lens allowed the research team to adopt an inductive approach to analysis because individual participant accounts can be considered as multiple truths. From this, a coherent narrative can be constructed to represent the collective truth of the final sample. These respective approaches methodologically underpinned the decision to analyse interview transcripts using inductive thematic analysis [38,39]. Analysis was supported by NVivo 12 software.

Analysis followed six systematic stages: data familiarisation; generation of initial codes; identification of themes; review and revision of thematic structure; defining final themes; and report writing [LJ]. Data saturation was achieved after analysing seven transcripts, though recruitment continued until the target sample size had been reached. Final themes were named based on the principal investigator’s interpretation of participant experiences [LJ]. Theme names were determined as best representing the essence of identified themes, which were verified by the rigorous and systematic approach to analysis [41,42]. Additionally, an iterative approach was taken whereby earlier stages of analysis were revisited after themes had been named to ensure that conceptualisations were grounded in the accounts of participants, as opposed to the researcher’s pre-conceived ideas and expectations. Finally, the analysis was consultative, meaning that final themes and theme names were agreed upon by all members of the research team as best representing the lived experiences of the study sample [EO, JH, RG, and AH]. The appropriateness of theme names is supported by an abundance of relevant participant quotations and rich conceptual interpretation and description. Research participants were not consulted during data analysis.

## 3. Results

The current study aimed to explore the lived experiences of parenting-specific guilt and shame in the first 16 weeks postpartum. A total of 20 women participated, of 23 who were approached (8% attrition), which is typical of perinatal recruitment [43]. Reasons for dropout included hospitalisation of an infant (one participant) and failure to respond to three contact attempts (one participant). The remaining individual was deemed ineligible at initial contact, due to infant age exceeding 16 weeks. The ineligible mother was verbally and electronically debriefed. During this process, participants were made aware that they could request a short 2-page summary and/or a copy of any final written reports from the current study by emailing the principal investigator [LJ].

Maternal age ranged from 25 to 35 (MAge = 30.71). Most women were married (57.14%), primipara (71.43%), and undergraduate educated or above (65%). Nearly two-thirds of those interviewed were in an [associate] professional occupation (60%), and the remaining were from caring, leisure, and other service occupations (20%); sales and customer service occupations (5%); unemployed (5%); administrative and secretarial occupations (5%); and managers, directors, and senior officials (5%). Participants self-identified as White British (35%), White European (15%), British Pakistani (10%), White Mixed Caribbean (5%), British Indian Asian (5%), Bangladeshi (5%), and Asian British Bangladeshi (5%). Infant age ranged from 4 to 16 weeks (Mean age = 9.6). There was an exact 50:50 split between mothers interviewed whose infant was between 4 and 8 weeks old, and whose infant was between 12 and 16 weeks old. There was also an exact 50:50 split in infant sex (M–F).; see Table 1 for participant demographic characteristics.

Thematic analysis identified three major themes, each with two sub-themes: ‘No man’s land—no longer a woman, not feeling like a mum’ (sub-themes: ‘Mum gets left by the wayside’ and ‘Under the magnifying glass’), ‘Comparison is the thief of joy’ (sub-themes: ‘Body failure’ and ‘Feigning ‘fine’), and ‘Protecting against the unrelenting storm’ (sub-themes: ‘Pouring from an overflowing cup’, and ‘The polarising role of social media in motherhood’). The final thematic summary can be found in Table 2.

### 3.1. Theme One: No Man’s Land—No Longer a Woman, Not Feeling Like a Mum

Mothers felt guilty for neglecting self-care but equally felt guilty when prioritising themselves. The latter was due to beliefs held that the mother was neglecting her parenting duties in putting herself first, embodying the mothering myth. Participants also struggled with feeling deprioritised by their healthcare and social support networks, which at times led to resentment being held towards one’s infant. Romantic relationship strain and ruminating about potential infant harm, consequential of parental (in)action, also triggered guilt and shame. Reframing one’s actions as being in the infant’s best interest, on the other hand, protects against these feelings.

#### 3.1.1. Sub-Theme One: Mum Gets Left by the Wayside

New mothers were consumed by guilt for wanting to engage in self-care. This was interpreted as deviating from the motherhood myth of needing to prioritise one’s infant at all costs:

*I’m just constantly worried about like a variety of things and if I meet up with my friends for coffee- that’s not an experience for the baby so should I always be going somewhere that baby’s gonna get benefit from.*

(Galinda)

This left participants in a precarious position because neglecting one’s needs also resulted in guilt:

*You feel guilty in yourself in terms of not giving yourself enough time. Enough self-care… I’m always putting my health to one side to sort of look after the children and it does get on top of you.*

(Daisy)

Feeling deprioritised was also evoked externally. Many participants felt that their mental health was marginalised during routine postpartum appointments and by their social support network:

*I think mental health gets a bit of a tick box [from midwives and health visitors], like you kind of get asked like, ‘…and how are you doing in terms of anxiety’…If I had been having a harder time with it, I don’t think the way that I’ve been asked about how my mental health is would have necessarily encouraged me to share that information.*

(Ashley)

*I saw everyone just shower him [baby] with so much love but forgotten me, and I was like. ‘Well, he’s only here because I’m here’, so I had those initial [guilty] feelings…*

(Elsie)

Unrelenting parenting responsibilities inflamed guilt and shame. At times, this caused resentment to be held towards one’s infant:

*…not like being able to keep the house as tidy or, you know, give your children enough time, give your husband enough time…not being able to like, you know, look after yourself. That could bring about shame as well, right? Your anger- frustration that you can just split yourself into pieces and be able to do it all.*

(Veronica)

*To be honest, sometimes I can be a little resentful towards [baby] because he does take up so much of my time and needs so much…that’s what I think about feeling guilty.*

(Eleanor)

Guilt was also consequential in relationship strain among those interviewed:

*[I question] why is he [husband]- wasn’t washing something up, or, you know, just just the silly little things…and I feel bad for doing that.*

(Josie)

#### 3.1.2. Sub-Theme Two: Under the Magnifying Glass

New mums were vulnerable to self-blame for their infant’s development and health status. This sometimes caused dissociation from the maternal identity:

*I do feel a bit ashamed when I do go out and see other children of similar age because I’m thinking is it my fault again, you know what I mean? Is it because I’m not a good enough mum? I haven’t pushed her, you know what I mean… like I haven’t given her enough time and sat with her…*

(Daisy)

Questioning one’s parenting (in)actions was intrinsically tied with empathic concern for infant wellbeing, perceived inadequacy, and rumination about one’s suitability as a mother. Infant feeding was a dominant manifestation of these ruminations:

*You feel as though you’re neglecting the child, because you’re like, ‘Hang on a minute, if I haven’t fed her for three hours, if I haven’t changed this…er, am I not good enough for her? Would she be better off with someone else?’, sort of thing, ‘Should I be a mother?’.*

(Betty)

*I know everyone is different…but you know we do have quite strong wishes like I don’t want to pop into McDonalds when he’s a toddler, I just really want him on the best food we can give him and that starts with breastfeeding for me.*

(Lola)

However, reframing one’s actions as being in their infant’s best interest protected the *‘good mother’* identity:

*I felt bad giving her formula but on the side of it I could see how much more content she was from having a good full feed and that’s what made me kind of overcome the guilt.*

(Eponine)

Feeling unable to make one’s baby happy also caused distress and concern about one’s suitability as a mother:

*If they’re [baby is] upset and you don’t know what they want, and you feel ashamed cause you’re like ‘what do they want?’*

(Jane)

Feeling negatively judged for one’s parenting not only resulted in feeling the need to defend one’s actions but also led to the avoidance of situations that could potentially elicit these emotions:

*I do get a bit defensive like someone has kind of criticised me for not having enough wear on the baby on the bus the other day and I think I said, ‘Well, we are from [country], she is fine. We are from a very cold climate’ erm so I guess I felt a little bit of shame.*

(Clarabella)

*Women who go to those [breastfeeding] groups are exclusively breastfeeding whereas I’m at the minute 99% bottle feeding and trying to move to breastfeeding. So that’s the reason I don’t feel comfortable really going there, and yeah, I do feel like I would be judged for bottle feeding even when I’m desperate to breastfeed.*

(Lola)

### 3.2. Theme Two: Comparison Is the Thief of Joy

Restrictions on movement when recovering from birth and struggling with changes to one’s postpartum appearance both triggered guilt and shame. Psychologically, mothers felt inadequate and ungrateful if they held negative emotions about new motherhood or if they faced parenting challenges. Making comparisons with the developmental milestones of other infants was also a source of guilt and shame. Most participants felt unable to confide in others due to fears about being judged as not coping with motherhood. This was uniquely tied to shame and avoidance behaviour.

#### 3.2.1. Sub-Theme One: Body Failure

Mothers experienced guilt when they were unable to carry out household and childcare tasks due to physical restraints. Participants were plagued by beliefs that they *‘should’* have been able to undertake normative tasks and often felt like a burden to their loved ones:

*I went to stay at my mums for 2 weeks [after being discharged from hospital] because of how poorly I was so I could have help with the first born…but the very next day I woke up and I felt guilty of like everyone sort of having to run around and, you know, look after my kids…*

(Daisy)

Caesarean section recovery was prominent in the accounts of women who were experiencing guilt. This also instilled shame and hopelessness when participants believed themselves to be abandoning their child or shunting their maternal responsibilities:

*I’d look at him [baby] and I couldn’t bend, and I couldn’t do anything, and obviously looking at him [husband] and showering him [baby] with so much love…that triggered me a lot.*

(Elsie)

*My close friend she’s just had her 4th and… she’s just not able to like pick him [baby] up as much because she’s had a caesarean… she feels really ashamed that she’s not really like there for her child. Not being able to pick him up…especially when he’s like crying out for her…*

(Veronica)

Another cause for distress included postpartum body image adjustments:

*I used to be a size 8 or a 10 and became a 16 to 18 [during pregnancy] and I just felt like I ballooned out. I had a small torso and just feeling so huge really affected me. He was 8 pound 4 and quite a long baby and I just think back and wonder how he fit inside of me. But seeing my body change like that, was shocking for me.*

(Eleanor)

This, in part, stemmed from internalised societal expectations to quickly *‘bounce back’* to one’s pre-pregnancy weight and level of fitness after giving birth:

*They say you bounce back, or some people bounce back, but that wasn’t the case for me. After I gave birth, I still had a belly where I looked like I was pregnant, and I was quite ashamed of myself like why have I not bounced back? And I still put so much pressure on myself.*

(Elsie)

This was worsened by insensitive comments received by others regarding one’s size:

*Even if people are being quite nice, I think it’s just not a nice feeling that you think it’s okay to just talk about my body openly. I kind of feel very insecure.*

(Siobhan)

#### 3.2.2. Sub-Theme Two: Feigning ‘Fine’

Comparing one’s baby with the developmental milestones of other children triggered feelings of failure. This was true for mothers making both upwards and downwards comparisons:

*There is shame and guilt sometimes when your baby is doing better than average…when people say to you like. ‘Oh, your baby has so much hair, they look so much more alert, and ‘they are already rolling’ or whatever, when their baby is just starting to roll-. I’m like, ‘Oh you know, she’s- I just give a joke or say I don’t know. I downplay it all.*

(Clarabella)

All participants referred to rose-tinted and unattainable societal depictions of new motherhood. Failing to acknowledge negative experiences triggered guilt and shame, because participants believed themselves to be deviating from accepted narratives:

*Society puts such a spin on [parenthood] that it’s so perfect and you’re so blessed, and you are in a lot of ways. But there is such a spin on it that if you dare feel anything that deviates from that narrative, it’s like, ‘You knew what you were in for’ [when you chose to have a baby].*

(Ophelia)

In extreme cases, feelings of overwhelm contributed to depressive symptoms and suicidal thoughts:

I feel hopeless. Other times I just feel drained and not good enough. I have in the past had a little thought like ‘why am I even here’…I just thought f*** it, I’ve had enough, I can’t carry on.

(Eleanor)

Among those interviewed, shame was linked with avoidance of triggering scenarios and self-isolation from one’s social network. This perpetuated mental health difficulties because participants felt the need to give the impression that they were coping better than they were, for fear of judgement:

*With other problems I would turn to my mum, but…I don’t want mum to think, ‘Oh she can’t manage it, she can’t handle it, she needs me to step in’ or things like that. So, no, really, I haven’t really turned to anybody this time.*

(Galinda)

Grieving one’s pre-pregnancy life was commonplace but was equally closely intertwined with guilt, due to downwards comparisons made with other women. In this respect, women feared that they were being ungrateful:

*You grieve for, you know, almost overnight, you’ve been handed this baby that just wants, you know, just wants all of you and you just want five minutes, and you don’t necessarily get that because you’re always thinking about the baby, and then you feel terrible…you feel guilty because there’s many women out there that can’t have babies.*

(Heather)

### 3.3. Theme Three: Protecting Against the Unrelenting Storm

Practicing grounding techniques allowed participants to become more self-compassionate. Re-framing parenting expectations to be more manageable, celebrating parenting successes, and protecting time for self-care, all nurtured postpartum wellbeing. Likewise, confiding in those with shared life experiences empowered mothers to persevere through difficulties. Social media was a useful facilitative tool in this support-seeking process, although there was recognition among participants that social media should be used responsibly. Social media could also perpetuate the internalisation of competitive and consumerist-driven parenting beliefs, which exacerbated guilt and shame.

#### 3.3.1. Sub-Theme One: Self-Compassion and Pouring from an Overflowing Cup

Being curious and non-judgemental about intrusive feelings enabled participants to be more self-compassionate:

*I’m kind of just deciding what is the truth, like, ‘Should I feel guilty? Is there a real like reason to feel that way?’ I’m just kind of analysing my behaviour and if there’s a real reason to feel guilty.*

(Gloria)

Slowing down and grounding oneself in the present also dissipated guilt and shame:

*If I do look at someone and I think that they are judging me for something or thinking I shouldn’t have done that or something, I always just look at my baby, I think for me she is an anchor that brings me totally back down to earth…*

(Henrietta)

Practicing more realistic parenting standards and protecting time for self-care also alleviated parenting pressures:

*Make the child work around you rather than the other way round…it’s okay for [baby] to cry. You don’t have to tend to him straight away, like, if I’m eating and he’s crying, I’ll finish off a couple of bites…it’s fine for a child to cry, they’re meant to cry…look after yourself as well I’d say.*

(Eleanor)

Sharing one’s experiences with other new mothers normalised and validated one’s difficulties and alleviated moral distress:

*I think my friend, she has kids similar age and she’s going through the same sort of thing. And if we are out together, I don’t feel as bad as we’re um both in the same boat.*

(Ronnette)

Making light of one’s parental challenges and focusing on achievements diminished the emotional sting and protected against guilt and shame:

*I think my husband- cos I think we can try and keep it into perspective, and then maybe even laugh about it, but you know, if baby girl does cry all afternoon, it doesn’t mean that we are doing anything wrong.*

(Clarabella)

#### 3.3.2. Sub-Theme Two: The Polarising Role of Social Media in Motherhood

For some, confiding in those with shared life experiences alleviated guilt and shame:

*…I’ve seen other women and mothers going through the same thing [on Peanut app] …that’s only, I would say the only thing that’s helping in the sense that I’m not alone.*

(Josie)

Social media was outlined as a positive tool for connecting with other mothers and for empowering oneself. Still, recognition was made that social media needed to be used responsibly to be a useful mental health tool:

*I’m quite active on social media. So, like on Insta, I vlog my life and what not. So, through that I think those, that has opened doors for me, like, I’ve had loads of friends from like my school, college, Uni, and they’ve reached out to me saying like, ‘Oh my god. I’ve had a baby too, like, let’s hang out’…through social media I’ve been able to do that.*

(Elsie)

*I’m not saying that everything that you see on social media is positive, but…you just focus on the posts that you want to pay attention to, don’t you?*

(Eponine)

Although social media played an unequivocally positive role in enhancing social connections and protecting against guilt and shame, it was recognised that social media could also perpetuate competitive attitudes towards mothering, if not navigated carefully:

*I think social media and just the kind of amount of information that’s out there about how to raise children or babies can put a lot of pressure on new mums, that you should always be doing something with- like if, if at any point in the day you’re just kind of sat watching TV or trying to have like a meal, then you’re not doing enough kind of thing.*

(Ashley)

Concerningly, social media was perceived as promoting consumerist ideals, which also triggered guilt and shame:

*With the cost of living at the moment as well, and money and maternity pay isn’t amazing, it’s- I’m constantly feeling guilty that I’ve not bought him the newest sensory book, or he’s not got the best playmat, or he doesn’t go to a class every day of the week…I had to buy some things second hand…he’s not having the same experience as the children I’ve seen on Instagram*.

(Galinda)

Finally, unrealistic social media posting can also trigger perceived inadequacies about one’s postpartum body:

*…it makes you feel like that mum looked great after having baby, or she looks like she can go and do loads of things, when actually, that’s not real.*

(Eponine)

Failing to meet unattainable parenting standards resulted in shame and avoidance of potentially triggering situations:

*It might make you maybe resentful towards others who you think aren’t feeling those things or are meeting the expectations that you want to be meeting…not wanting to interact with people who you think aren’t feeling those things, so it provably isolates you and perpetuates the feeling.*

(Ophelia)

Concerns were raised among participants that societal depictions of *‘good mothering’* were so varied and at times, contradicting, that women were literally unable to tend to everyone’s wishes:

*Everyone’s got advice and if you took it all on board, you’d just be doing everything and doing the opposite of everything, if that makes sense.*

(Ashley)

## 4. Discussion

The current study used inductive thematic analysis to explore the lived experiences of parenting-specific guilt and shame in the first 16 weeks postpartum. Participants struggled to navigate the conflict between meeting their parenting responsibilities and needing respite. This is a manifestation of intensive mothering theory [19,20], whereby good mothering is attributed to childrearing, which involves high resource investment and the romanticisation of self-sacrifice. Deviating from intensive mothering ideals drove postpartum emotional distress among those interviewed. To protect the good mother identity, significant re-framing of one’s parenting (in)actions is necessary [44]. The same was true in the current study, whereby reframing protected against guilt and shame. Based on this collective evidence, future research should prioritise the development of interventions, which are designed to target a harsh inner critic, for example, self-compassion, positive reframing, or mindfulness-based interventions [45,46].

Rumination [47] and counterfactual thinking [48] cognitively underlie guilt and shame. Reflected in the current study, mentally undoing one’s parenting (in)actions was characteristic of postpartum guilt and shame. Unique to shame, this resulted in dissociation from one’s maternal identity and avoidance of potentially triggering situations. Both emotions were, however, intrinsically tied to empathic concern for infant wellbeing. Interventions designed to address biased cognitive appraisals, e.g., Cognitive Behavioural Therapy may hold utility for re-framing self-critical thinking around one’s parenting practices [49,50].

Feeling deprioritised by one’s healthcare team and social support network also exacerbated guilt and shame. The protective effects of well-perceived support from one’s intimate partner [51] and healthcare professionals [52] are well documented. Feeling ill-supported in the current study, resulted in resentment being held towards one’s infant. This is problematic because infant resentment has been linked with childhood behavioural issues [53]. Current findings therefore mirror previous calls to strengthen the involvement of intimate partners in routine appointments (to give parity of esteem to paternal and maternal mental health screening and support) and to invest in resources and specialist training that will enable healthcare practitioners to deliver this type of necessary holistic support [54].

Physical recovery from childbirth, and particularly from a caesarean section, was a common trigger of guilt and shame. Previous research has identified elevated depression scores among those who had an unplanned caesarean section, when compared with an elective caesarean section [55]. Likewise, quality of life is reportedly higher [56], and physical complaints lower [57] among those who had a vaginal birth when compared with caesarean section delivery. Current findings suggest that restrictions on one’s physical ability to fulfil childcare responsibilities and feeling dependent on others during birth-recovery might be pivotal intermediaries between mode of delivery, guilt, shame, and postpartum depressive symptoms. To the first author’s knowledge, there have been no previous attempts to quantitatively map these relationships, identifying a new line of research inquiry.

In the current study, participants struggled to navigate internalised societal pressures to bounce back to their pre-pregnancy weight, consistent with previous literature [58]. A staggering 68.8% of women experience body dissatisfaction after childbirth [59], which is persistent following weight loss efforts [60]. Poor body image is significantly associated with depressive, but not anxious, symptomatology in the early postpartum [61]. This is further aggravated by unsolicited comments made by others [62]. Focusing on body functionality and acceptance can combat unrealistic postpartum body image ideals [63]. Current findings reinforce pre-existing literature and mirror recommendations to develop re-framing interventions, with an aim to protect postpartum emotional wellbeing. Educating mental health practitioners on common postpartum body image concerns is also paramount for addressing body image concerns and for protecting healthy eating practices [64].

Participants noted that depictions of new motherhood were often rose-tinted, which created conflict when negative feelings about motherhood were experienced. This might be explained by ideal–actual discrepancies [65]. Current findings argue that there is a pressing need to construct more realistic narratives of what it is to be a good mother, which allow negative and positive feelings to be held, simultaneously [19]. Those interviewed were hesitant to confide in others about parenting challenges, due to fears of being judged as an incompetent mother. This was uniquely linked with shame and avoidance of potentially triggering situations. National campaigns can effectively address problematic public attitudes, for example, mental health stigmas [66]. Similarly, strengthening the maternal social support network, e.g., through interventions and via inclusion in routine perinatal appointments, has a positive impact on postpartum mental health outcomes [67,68]. Developing multifaceted solutions in this manner may aid in normalising the discussion of postpartum transitional difficulties.

Social media was a useful tool for support-seeking, although there was recognition that it could also perpetuate competitive parenting practices. In the current study, women presented consumerist-driven parenting beliefs [69]. Specifically, participants likened purchasing the most expensive available formula milk with optimal parenting, to morally off-set the decision not to breastfeed [70]. Other participants experienced guilt and shame for purchasing second-hand baby items, being unable to compete with the lifestyles of celebrity parents, making comparisons with the developmental milestones of other infants, and comparing one’s postpartum body against those of celebrity parents. The current study sits within the context of the UK cost of living crisis [71], which has had dire consequences for rates of poverty, living standards, and prevalence of mental distress [72]. Current findings suggest that financial strain imposed by this broader societal context may have magnified inequalities regarding postpartum guilt and shame. Although further research is required to corroborate this evidence, providing guidance on mindful technology use during routine perinatal appointments is also recommended. This can be facilitated by signposting mothers to reputable organisations for medical and mental health information, e.g., the Royal College of Obstetrics and Gynaecology (RCOG), and by enhancing maternal self-esteem so to combat impulses to engage in upward comparisons when seeking online parenting support [73]. This may be achieved by encouraging mothers and their support persons to celebrate parenting successes, to re-frame harsh automatic negative thoughts, and to protect time for self-care [74].

### Strengths, Limitations, and Future Directions

To the first authors’ knowledge, this study was the first to explore lived experiences of postpartum guilt and shame, extending pre-existing infant feeding research [16]. By better understanding the nuances of guilt and shame from the perspective of new mothers, a more accurate measurement of these emotions may be achieved. Conceptualisations of postpartum guilt and shame in the current study can be used as a springboard for comparisons against other perinatal populations, e.g., pregnancy-specific and birth-specific guilt and shame. The final sample was equally distributed in terms of infant sex and age, and the final sample represented diverse ethnicities. This is especially poignant because traditionally, women from non-White backgrounds are underrepresented in perinatal research [23]. The current study, therefore, contributes novel insights to the literature base. However, diversity was not achieved with respect to educational or occupational status or with regard to maternal parity. As such, some caution should still be reserved when interpreting current findings. Demographic questions were inconsistently recorded among student investigators during the process of interviewing, leading to some missing reporting in Table 1. As such, some reservations should be made regarding the transferability of study findings. This may be resolved by ensuring that one researcher conducts all interviews for consistency, by training junior members of the research team on the importance of standardisation during data collection, and/or by member-checking transcripts after transcription for missing information and troubleshooting issues earlier in the process of recruitment and data collection.

Mode of birth delivery was not routinely captured in the current study. For those who spontaneously recalled their birth experience, this had had a marked effect on their practical parenting capabilities and on their experiences of guilt and shame. In pre-existing literature, caesarean birth significantly predicts posttraumatic stress symptoms [75]. As such, this spontaneous factor may have inadvertently confounded reported experiences. Body Mass Index, too, was not routinely recorded yet was a spontaneously reported. Perinatal women who have overweight or obesity are at elevated risk of depression, anxiety, eating disorders, and bipolar disorder [76]. The current study aligned with previous literature that exposure to thin ideals on social media exacerbated postpartum guilt and shame [77,78]. Future research should consider a dedicated investigation of the relationship between birth experiences, Body Mass Index, and postpartum guilt and shame. In recognition of critical developmental milestones inherent to the first 1001 critical days [79], a longitudinal mixed-methods investigation is recommended to more deeply understand the transitional needs of new mothers in the first two years postpartum.

Interview schedule questions did not cover the quality of the mother’s relationship with her romantic partner [80], wider family [81], healthcare team [82], or the potential role of the workplace [83] in her experience of postpartum guilt and shame in the first 16 weeks postpartum. The interview schedule was intentionally designed to be exploratory and broad-scoping. This decision was made to allow participants to focus on topics salient to their personal experiences, without imposing pre-conceived ideas and expectations from the research team, based on a prior understanding of the literature base. However, given the importance of the wider sociocultural context in contributing to maternal guilt and shame [84], capturing these experiential accounts would have further enhanced the richness of data collected from participants. Future research should seek to explore lived experiences of these broader contextual factors and how these contribute to experiential guilt and shame in the first 16 weeks postpartum.

The number of interviews conducted via telephone versus video conference calling software was not routinely collected during the recruitment process. This is problematic because there are respective strengths and limitations to both modes of data collection [85,86]. Telephone interviewing is particularly problematic as disrupted visual cues (inherent to video conference calling interviews) may open data collection to more potential misinterpretation [86]. In the first author’s 10 years of experience, however, telephone interviews are particularly favoured among perinatal populations. This may be due to their flexibility and enabled mobility (relative to video conference calling software), allowing them to engage in childcare responsibilities while engaging in the interview process. Further to capturing this information routinely in future research, conducting a methodological study that is dedicated to investigating the relative utility of video conference calling software and telephone interviewing, and is specific to perinatal populations, would be an invaluable contribution to the literature base. Finally, participants were not given the opportunity to comment on their transcript after the interview had taken place. In retrospect, giving the opportunity to member-check transcription would have improved the trustworthiness of the data collected [87]. Future research should ensure ample opportunity for participants to check their transcript prior to analysis, in a reasonable timeframe to ensure that transcription is accurate and reflective of participant accounts.

## Figures and Tables

**Table 1:** Participant demographic characteristics.

Participant Pseudonym	Age	Infant Age/Weeks	Parity	Infant Sex	Highest Level Education	Occupation	Marital Status	Ethnicity	Relevant Contextual Factors
Eleanor	35	4	Primiparous	Male	Level 3	Caring, leisure, and other service occupations	Living with partner, Single	White British	
Lola	29	6	Primiparous	Male	Level 2	Sales and customer service occupations	Single, Living with partner	White European	
Clarabella	31	8	Primiparous	Female	Level 7	Professional	Married	N/A *	
Ronnette	29	15	Multiparous	Male	Level 2	Associate professional	Civil partnership	White British	
Heather	33	6	Primiparous	Male	N/A *	Professional	Married	White Mixed Caribbean	Traumatic birth experience, Preeclampsia diagnosis.
Lucille	25	16	Primiparous	Female	Level 3	Unemployed	Single	White European	
Cecilia	33	12	Primiparous	Male	Level 7	Professional	Living with partner, Single	White British	BMI above 25 during pregnancy, Difficult C-section recovery.
Gloria	35	6	Primiparous	Female	Level 6	Professional	Married	White European	
Ophelia	30	8	Primiparous	Female	Level 7	Professional	Cohabiting, Engaged	White British	
Henrietta	32	12	Multiparous	Female	Level 2	Managers, directors, and senior officials	Married	N/A *	
Elsie	30	15	Primiparous	Male	Level 6	Professional	Married	British Indian Asian	
Siobhan	34	12	Multiparous	Female	Level 7	Professional	Married	N/A *	
Éponine	34	9	Primiparous	Female	Level 7	Professional	Single	N/A *	
Daisy	29	12	Multiparous	Female	Level 6	Professional	Married	British Pakistani	
Betty	29	6	Primiparous	Female	Level 7	Caring, leisure, and other service occupations	Married	Bangladeshi	Traumatic birth experience, C-section complications
Galinda	28	7	Multiparous	Male	Level 6	Caring, leisure, and other service occupations	Married	White British	
Jane	23	15	Multiparous	Male	Level 3	Administrative and secretarial occupations	Single	White British	
Veronica	36	12	Multiparous	Male	Level 7	Professional	Married	British Pakistani	
Josie	37	16	Primiparous	Female	Level 7	Caring, leisure, and other service occupations	Married	Asian British, Bangladeshi	Cow’s milk protein allergy
Ashley	30	6	Primiparous	Male	Level 6	Professional	Single, Cohabiting	White British	

* Please note: Due to inconsistencies in the demographic questions asked during interviews [EO, JH, RG and AH], the flagged cells are unpopulated due to this information not being routinely collected.

**Table 2:** Thematic analysis: final structure summary.

Major Theme	Sub-Theme
No man’s land—no longer a woman, not feeling like a mum	Mum gets left by the wayside
	Under the magnifying glass
Comparison is the thief of joy	Body failure
	Feigning ‘fine’
Protecting against the unrelenting storm	Pouring from an overflowing cup
	The polarising role of social media in motherhood

## Data Availability

Front-facing participant documentation and anonymised interview transcripts can be made available on reasonable request to the corresponding author (leanne.jackson@liverpool.ac.uk). Anonymised interview transcripts are available through the data-sharing repository, Dryad (https://datadryad.org/stash) as of 3 December 2024. Raw audio files were destroyed two weeks after each interview took place to protect participant identity and thus are not available for re-use. Participants’ contact details are destroyed and thus are not available for re-use.

## Appendix A. Interview Schedule

Thank you for agreeing to talk to me today about your experiences of new motherhood. We are interested in your own personal experience, which may be different from other people—so there are no right or wrong answers, and you will not be judged based on what you say.

We would like to record the conversation with your permission.

We will be able to arrange an opportunity for you to see the transcript.

Should you wish to stop the interview at any time, or take a break, please tell me.

Also, you do not have to answer any question(s) that you do not feel comfortable answering.

Our discussions will remain confidential, and all data will be anonymised (see information sheet).

This interview is split into five sub-sections. Firstly, you will be asked some demographic questions, this is so that we can describe our sample. In section two, you will be asked how you define guilt and shame, and how these feelings are similar and different at this stage of motherhood. If, for you, guilt and shame are the same or too difficult to separate, this is fine as well. There are no right or wrong answers when answering these questions, we are entirely interested in your thoughts, experiences, and opinions. In sections three and four, you will be asked a broad question about the main forms of guilt, and shame, respectively, which are felt at this stage in motherhood. Here we may ask you some follow-up questions, this is not because you have not given us enough information or because you have not responded well enough, but rather it is so that we can make sure that we have covered all bases, to make sure that you have an open platform to talk about all aspects of new motherhood, which are important to you. In section five, you will then be given the opportunity to talk about anything that we may have missed in other sections of this interview.

Do you have any questions?

With your consent, I will now start the audio recorder and take your verbal consent.

[Take audio-recorded, verbal consent]

1. **Demographic information**
  - What is your age?
  - How old is your youngest baby (in weeks)?
  - What is your highest level of education?
  - What is your occupation?
  - What is your marital status?
  - What is your ethnicity?
2. **Definitions**
  - How would you define guilt and shame?
  - To you, what are the key similarities and differences between guilt and shame?
    ○ Feelings?
    ○ Thoughts?
    ○ Behaviours?
    ○ Interactions with others?
    ○ Which plays a bigger part in your current stage of motherhood?
    ○ Do they feel different? Have different effects? Stay for different amounts of time?
3. **Guilt**
  - What are the main forms of guilt that women have at this stage of motherhood?
    ○ Day-to-day activities? [routine with baby, e.g., feeding, playing, out and about, in healthcare settings, at night, relationships with others, relationship with yourself]
    ○ Feelings? [feelings that trigger, feelings that occur as a result]
    ○ Thoughts? [thoughts that trigger, thoughts that occur as a result]
    ○ Behaviours? [behaviours that trigger, behaviours that occur as a result]
  - What do you usually do when you feel guilty?
    ○ People you turn to?
    ○ Thoughts and/or behaviours that worsen?
    ○ Thoughts and/or behaviours that soothe?
  - For you, how impactful is guilt in your experience of motherhood?
    ○ How often do you experience this emotion?
    ○ Are there other feelings that are more important? Less important?
4. **Shame**
  - What are the main forms of shame that women have at this stage of motherhood? Day-to-day activities? [routine with baby, e.g., feeding, playing, out and about, in healthcare settings, at night, relationships with others, relationship with yourself]
    ○ Feelings? [feelings that trigger, feelings that occur as a result]
    ○ Thoughts? [thoughts that trigger, thoughts that occur as a result]
    ○ Behaviours? [behaviours that trigger, behaviours that occur as a result]
  - What do you usually do when you feel ashamed?
    ○ People you turn to?
    ○ Thoughts and/or behaviours that worsen?
    ○ Thoughts and/or behaviours that soothe?
  - For you, how impactful is shame in your experience of motherhood?
    ○ How often do you experience this emotion?
    ○ Are there other feelings that are more important? Less important?
  - Anything else
    ○ Before we finish up, is there anything else that you would like to talk about, or have I forgotten to ask anything?

Thank you for your time. We will leave you with a list of organisations that you can contact if you need support.
